# Computationally efficient and sub-optimal trajectory planning framework based on trajectory-quality growth rate analysis

**DOI:** 10.3389/frobt.2022.994437

**Published:** 2022-10-28

**Authors:** Reiya Takemura, Genya Ishigami

**Affiliations:** Faculty of Science and Technology, Graduate School of Integrated Design Engineering, Keio University, Tokyo, Japan

**Keywords:** trajectory planning, planetary rover, computationally efficient, sub-optimal algorithm, RRT, anytime algorithm

## Abstract

A planetary exploration rover has been used for scientific missions or as a precursor for a future manned mission. The rover’s autonomous system is managed by a space-qualified, radiation-hardened onboard computer; hence, the processing performance for such a computer is strictly limited, owing to the limitation to power supply. Generally, a computationally efficient algorithm in the autonomous system is favorable. This study, therefore, presents a computationally efficient and sub-optimal trajectory planning framework for the rover. The framework exploits an incremental search algorithm, which can generate more optimal solutions as the number of iterations increases. Such an incremental search is subjected to the trade-off between trajectory optimality and computational burden. Therefore, we introduce the trajectory-quality growth rate (TQGR) to statistically analyze the relationship between trajectory optimality and computational cost. This analysis is conducted in several types of terrain, and the planning stop criterion is estimated. Furthermore, the relation between terrain features and the stop criterion is modeled offline by a machine learning technique. Then, using the criterion predicted by the model, the proposed framework appropriately interrupts the incremental search in online motion planning, resulting in a sub-optimal trajectory with less computational burden. Trajectory planning simulation in various real terrain data validates that the proposed framework can, on average, reduce the computational cost by 47.6% while maintaining 63.8% of trajectory optimality. Furthermore, the simulation result shows the proposed framework still performs well even though the planning stop criterion is not adequately predicted.

## 1 Introduction

In an extreme environment such as Mars or a volcanic area, mobile robots have been used in scientific missions or as precursors for a future manned mission. The robot calling a planetary exploration rover is mainly commanded from Earth with its autonomous mobility system. However, the communication latency between Earth and the rover often impedes the mission; therefore, autonomous mobility has been an essential system for the rover. The autonomous mobility system consists of the following three major processes: first, the rover recognizes an environment with its onboard cameras or light detection and ranging (LiDAR) sensors in order to create a geometrical terrain map. Subsequently, the rover plans a path or trajectory as motion set to reach a local goal specified on the map. Finally, the rover successively executes the planned motion based on its navigation and control subsystems. These processes are managed by a space-qualified, radiation-hardened onboard computer. The processing performance for such a computer is often limited, owing to the limitation to power supply. Hence, a computationally efficient algorithm in the autonomous system is favorable.

Motion planning is an important process for the rover to avoid many risks in challenging terrains, such as vehicle rollover on a sloped terrain, collision with obstacle rocks, or becoming stuck on wheels in loose soil. These issues are addressed by the grid-based path planning method (Carsten et al., 2007; Pivtoraiko et al., 2009; Ishigami et al., 2013). Most of the methods have been basically derived from a regular discretization of robot state space, such as state lattice. The grid-based planner generates a graph whose vertices are a discretized set of reachable states of the robot and whose edges are feasible motions. Then, an optimal path is calculated based on a cost function composed of several indices, such as the wheel slip in loose soil, posture angles on rough terrain, and path length. Here, a grid-based search algorithm is often used to find a path that provides the minimum value of the defined cost. However, the path is a resolution-optimal path, which largely depends on the resolution of the grid. Additionally, the computational effort of this approach often exponentially increases the dimension of the problem or the resolution of the grid.

As compared with the grid-based method, an incremental search using a random sampling method is often used to avoid requiring a discretization of the state space. For example, Rapidly exploring Random Trees (RRTs) have been widely applied in sampling-based motion planning of a mobile robot (LaValle and Kuffner, 1999). This method can efficiently find a sub-optimal solution even in high-dimensional planning problems, although it does not find the completely optimal solution. There are many related works focusing on the improvement of its optimality. An extension of the basic RRT toward an anytime algorithm can generate a more optimal solution as the number of iterations increases, where it evaluates whether a new path is more optimal than the previous ones (Ferguson and Stentz, 2006). The RRT* is also proposed as an asymptotically optimal planner that can guarantee the optimality of the path generated (Karaman and Frazzoli, 2011). Gammell et al. (2014, 2018) proposed a method that can improve the convergence rate and final solution optimality. In this method, once a path is found from the first trial, the method retries sampling only from the subset defined by an admissible heuristic to potentially improve the solution. A primary advantage of these algorithms is that they can solve the motion planning problem as long as they continue their incremental search.

With applications to planetary exploration, recent studies have addressed motion planning under uncertainty, which solves the planning problem under stochastic constraints (Ghosh et al., 2018; Inotsume et al., 2020; Mizuno and Kubota, 2020; Candela et al., 2022). These works plan robotic motions so that the probability of the worst-case situation is less than a specified tolerance, resulting in the robust path/trajectory generation for mobility risks. Although they contribute to safe and reliable robotic navigation, the issue of computational cost still remains when integrated with an incremental search algorithm.

Overall, the trade-off problem between the optimality/robustness of a solution and computational burden is still inevitable. Practically, the rover does not always need to obtain an optimal solution but a valid solution as quickly as possible. Although a compromise metric to determine the terminal point for the planning is developed (Hansen and Zilberstein, 2001), the relationship between terrain features and the metric is an open issue. Intuitively, challenging terrains are potentially less likely to improve the trajectory optimality than flat terrains, even if computational resources are used sufficiently. For each terrain type, the incremental search algorithm needs to stop its process appropriately while maintaining a certain degree of optimality.

This study aims to develop a computationally efficient and sub-optimal trajectory planning framework for a planetary exploration rover. We introduce the trajectory-quality growth rate (TQGR) to explicitly analyze the relationship between trajectory quality and computational cost. For each type of terrain, the TQGR is collected and statistically processed in an offline manner. The TQGR-based analysis can show an appropriate number of iterations that may potentially improve the trajectory optimality. Then, the analysis module is exploited for online trajectory planning; therefore, the proposed framework appropriately interrupts the incremental search and generates a sub-optimal trajectory for the rover with less computational burden. Numerical simulation studies are performed to validate the proposed framework.

The remaining of this article is organized as follows: Section 2 explains the trajectory planning framework with the TQGR-based analysis. Section 3 shows the trajectory planning algorithm with a quasi-dynamic vehicle model in loose and rough terrains. Section 4 shows the LiDAR-based terrain mapping system. Section 5 discusses the simulation results using the proposed framework. Section 6 draws the conclusion and presents a direction for future studies.

## 2 TQGR-based trajectory planning framework

### 2.1 Overview

**FIGURE 1:**
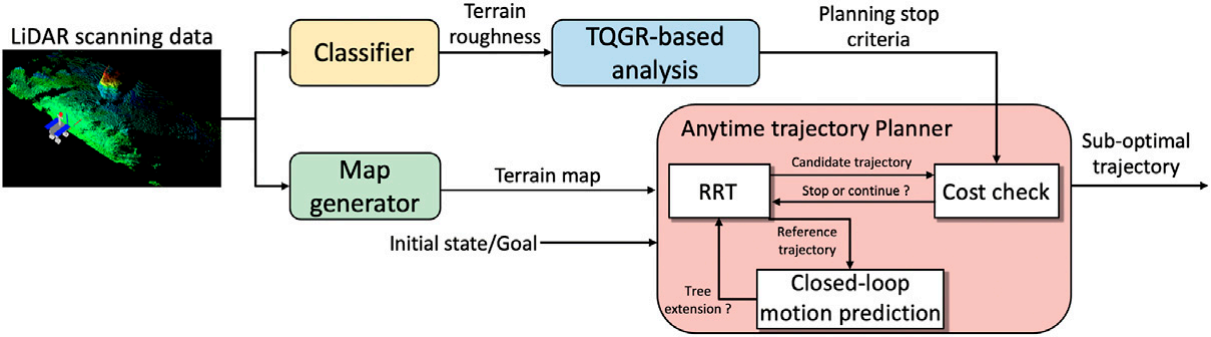
Proposed trajectory planning framework.

Figure 1 illustrates an overview of the proposed trajectory planning framework. The framework mainly consists of a map generator, an anytime trajectory planner, and a TQGR-based analysis module. The terrain map is generated by the conversion of LiDAR point cloud into a digital elevation map (DEM) as described in Ishigami et al. (2013). The scanned terrain data are also classified based on its roughness. The details are described in Section 4. The anytime trajectory planner employs our traversability-based trajectory planning (Takemura and Ishigami, 2021), where the closed-loop RRT (CL-RRT) (Kuwata et al., 2009) is used as an incremental search algorithm. The CL-RRT algorithm is suitable for a high-dimensional problem space being subjected to multiple constraints, such as robotic traversability and non-holonomic vehicle. Our previous work validated that the CL-RRT based on a quasi-dynamic vehicle model contributes to the reduction of vehicle slip risks in loose soil. Section 3 reemphasizes the detail of the trajectory planner. In this study, the CL-RRT is iteratively executed in an anytime approach to improve the trajectory quality defined by the cost function. The TQGR-based analysis module, which is statistically modeled offline, terminates the iteration of the CL-RRT. The following subsection explains the TQGR-based analysis.

### 2.2 Trajectory evaluation using TQGR-based analysis

**FIGURE 2:**
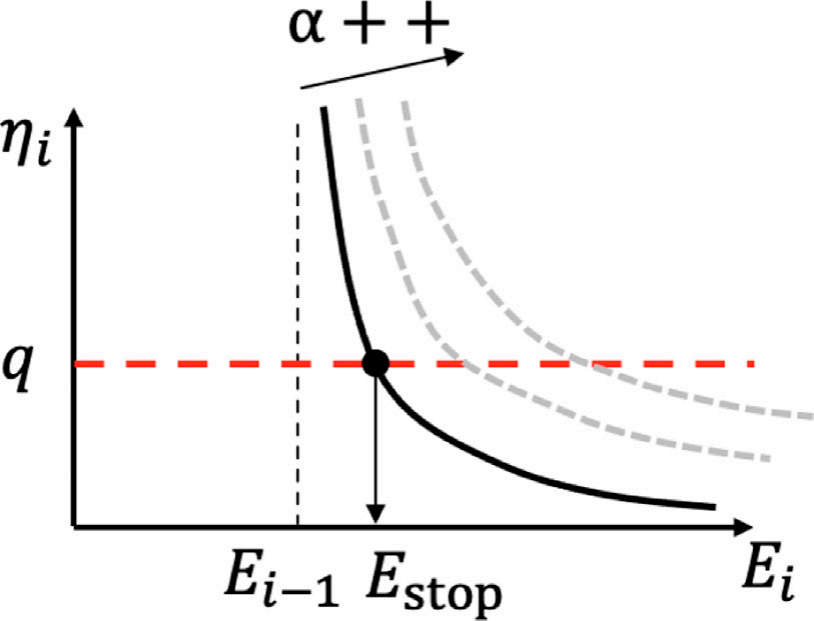
Intuition of the planning stop criterion (Eqs. 1, 2).

As proposed by Ferguson and Stentz (2006), the trajectory generated by the RRT converges to an optimal solution, but the number of iterations gradually increases. To appropriately terminate the iteration for each terrain type, the TQGR is introduced based on the trajectory cost and computational effort. When a new trajectory with less cost *C*_*i*_ is found at the *i*-th iteration, the TQGR *η*_*i*_ is defined as follows:

$$\eta_{i}=\frac{\left(C_{i-1}-C_{i}\right)/C_{i-1}}{\left(E_{i}-E_{i-1}\right)/E_{\text{max}}},$$ (1)

where *E* indicates the computational cost, which can be given by the calculation time or the number of sampling trials of the RRT algorithm. *E*_max_ is the maximum computational cost and is defined based on the number of iterations. The TQGR indicates the degree of improvement in trajectory quality relative to the computational cost. Then, the iteration for anytime planning is terminated when the TQGR at the *i*-th iteration is less than the expected value:

$$\eta_{i}<q,$$ (2)

where *q* is the planning stop criterion. Figure 2 depicts the intuition of the planning termination based on the TQGR and *q*. *q* can be given for each terrain type as follows:

$$q=f\left(M_{\text{label}}\right),$$ (3)

where $M_{\text{label}}$ is the classified terrain information. Eq. 2 indicates that the *i*-th iteration has a low expectation to improve the trajectory quality.

The function *f* (⋅) is modeled offline by the statistical analysis of the TQGR. In each type of terrain, the TQGR can be acquired at every iteration of the anytime planning. It should be noted that the trajectory planner is inherently subjected to the randomness of the RRT. To mitigate the randomness, the anytime planning is repeatedly executed, and, a series of TQGRs is calculated. The geometric mean of TQGRs is defined as the planning stop criterion:

$$q_{\text{label}}=m+1\sqrt{\eta_{0}\cdot \eta_{1}\cdot \cdot \cdot \eta_{m}},$$ (4)

where *m* is the number of TQGRs. Once *q*_label_ is calculated offline for each type of terrain such as flat, rocky, and slope, the TQGR-based analysis module can determine the feasible number of iterations for online planning. To simply model *f* (⋅), terrain roughness is used as $M_{\text{label}}$ , and the Gaussian process regression (GPR), which is one of the machine learning algorithms (Rasmussen and Williams, 2006), interpolates the dataset of $M_{\text{label}}$ and *q*_label_. This enables the TQGR-based trajectory planning framework to achieve a sub-optimal solution with a less computational burden even in unknown environments. Furthermore, to stop the planning before the trajectory with *C*_*i*_ is found at the *i*-th iteration, Eqs. 1, 2 are extended as follows:

$$\begin{array}{ccc} E_{i}>E_{i-1}+\frac{1-\alpha }{q}E_{\text{max}}, & & \\ \alpha =\frac{C_{i}}{C_{i-1}}, & & \end{array}$$ (5)

where *α* is the heuristic parameter, which shows the ratio between the possible cost *C*_*i*_ and the current cost *C*_*i*−1_. As shown in Figure 2, *E*_stop_ becomes large as *α* increases. The *α* assumption contributes to the appropriate termination of anytime planning, even during the *i*-th iteration. In practice, Eqs. 2, 5 are both used for online trajectory planning.

## 3 Anytime trajectory planner

### 3.1 Algorithm

The proposed anytime trajectory planner basically iterates a single procedure of the traversability-based CL-RRT (Takemura and Ishigami, 2021). The primary difference between the basic RRT and the anytime RRT is that the total cost *C*_*i*_ found at the *i*-th iteration is used for the tree expansion phase of the RRT at the next iteration phase. In order to find a new trajectory with a smaller cost than *C*_*i*−1_, the tree expansion phase at the *i*-th iteration should meet the following condition:

$$c_{\text{start},near}+c_{\text{near},new}+h_{\text{new},goal}<C_{i-1},$$ (6)

where *c*_start, near_ is the cost between the start state and the state nearest to the new state in the tree, *c*_near,new_ is the cost between the nearest state and the new state, and *h*_new, goal_ is the heuristic cost for the given goal area. In this article, the Euclidean distance is used for the heuristic cost. This cost assessment can exclude the useless tree extension, which leads to efficiently find a new trajectory with a smaller cost if it exists. The total cost exponentially decreases and converges to an optimal solution as the number of iterations increases.

Algorithm 1 Anytime trajectory planning.

**Input:** Goal region: *X*_goal_
**Input:** Terrain map: M

**Input:** Planning stop criterion: *q*
**Output:** Trajectory: $T_{\text{subopt}}$

1: *Y*_goal_ ←GetHeight $\left(X_{\text{goal}},M\right)$

2: *S* ← (*G*_*y*_, *G*_*σ*_) ←Initialize(***x***_init_, *Y*_goal_)
3: **while** i++ **do**
4: **while** 1 **do**
5: ***y***_rand_ ←Sample(*k*, *Y*_goal_)
6: $\left(G_{y_{\text{new}}},G_{\sigma_{\text{new}}}\right)\leftarrow$ Extend(*S*, ***y***_rand_)
7: **if** Traversability Assessment $\left(G_{\sigma_{\text{new}}}\right)$ and Check Eq. (6) **then**
8: *S* ←UpdateTuple $\left(S,G_{y_{\text{new}}},G_{\sigma_{\text{new}}}\right)$

9: **if** ReachGoal $\left(G_{\sigma_{\text{new}}}\right)$ **then**
10: T← GetTrajectory(*S*, *Y*_goal_)
11: **if** CostCheck (T) **then**
12: $T_{\text{subopt}}\leftarrow T$

13: *η*_*i*_ ←CalcTQGR $\left(T_{\text{subopt}}\right)$

14: break
15: **if** *η*_*i*_ < *q* **then**
16: break
17: Return $T_{\text{subopt}}$

**FIGURE 3:**
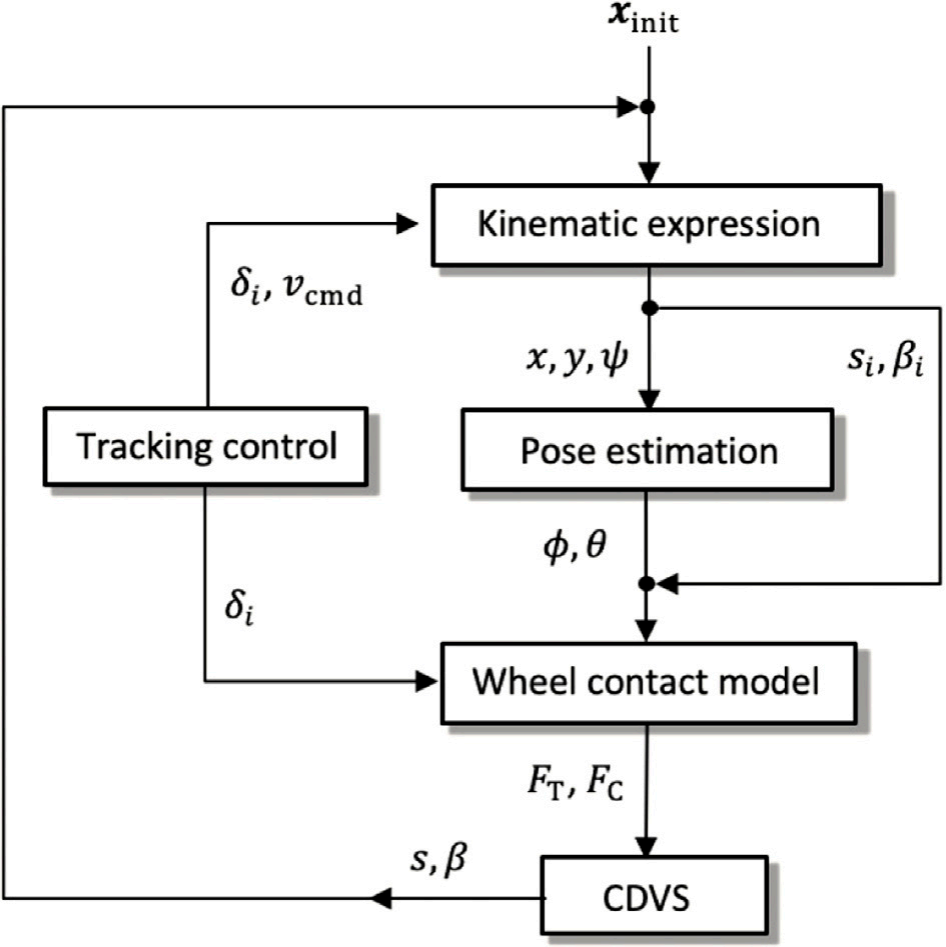
Consecutive calculation modules for the quasi-dynamic vehicle model.

Algorithm 1 shows the procedure of the anytime trajectory planner. Our trajectory planner incorporates the traversability assessment into the CL-RRT algorithm (Kuwata et al., 2009). Before starting the trajectory planning, GetHeight calculates the *z* coordinate for each goal position in *X*_goal_ using terrain information (Algorithm 1, Line 1). The sample function randomly samples a node ***y***_rand_ including x, y, and z coordinates (Algorithm 1, Line 5). The extend function returns two graphs: reference trajectories $G_{y_{\text{new}}}$ and state trajectories $G_{\sigma_{\text{new}}}$ (Algorithm 1, Line 6). As in Kuwata et al. (2009), the reference trajectories are extended toward the sampled node. The state trajectories are simulated by the quasi-dynamic vehicle model with the trajectory tracking controller (Figure 3). The quasi-dynamic vehicle model accurately predicts the robot motion and successively updates the state even in loose soil while tracking the reference trajectories. The traversability assessment examines the returned state trajectories to avoid mobility hazards for the rover on 2.5-dimensional rough and loose terrains (Algorithm 1, Line 7). Additionally, the constraint of the anytime approach in Eq. 6 is also checked. When the trajectories are traversable, the algorithm updates the tuple *S* (Algorithm 1, Line 8). The CostCheck function calculates the cost throughout the trajectory of reaching the goal (Algorithm 1, Line 11), considering the traversability index as follows:

$$\begin{array}{ccc} C_{i}=\int_{t_{\text{s}}}^{t_{\text{e}}}\left\{W_{\phi }{\left(\frac{\phi \left(t\right)}{N_{\phi }}\right)}^{2}+W_{\theta }{\left(\frac{\theta \left(t\right)}{N_{\theta }}\right)}^{2}\right. & & \\ \left.+W_{s}{\left(\frac{s\left(t\right)}{N_{s}}\right)}^{2}+W_{\beta }{\left(\frac{\beta \left(t\right)}{N_{\beta }}\right)}^{2}+W_{l}{\left(\frac{l\left(t\right)}{N_{l}}\right)}^{2}\right\}dt, & & \end{array}$$ (7)

where *i* indicates the *i*-th iteration, *t*_e_ − *t*_s_ is the elapsed time for a mobile robot to travel, *l* is the length of the trajectory segment, *ϕ* and *θ* are the roll and pitch angles of the robot, *s* is the slip ratio in the longitudinal direction of the robot, *β* is the sideslip angle of the robot, *N*_*l*_, *N*_*ϕ*_, *N*_*θ*_, *N*_*s*_, and *N*_*β*_ are the normalization factors that render each index dimensionless, and *W*_*l*_, *W*_*ϕ*_, *W*_*θ*_, *W*_*s*_, and *W*_*β*_ are the weighting factors that provide a specific priority for each index. In general, the weighting factors are user-defined parameters and constant throughout the trajectory planning. They are adjusted to have an equal influence on the cost function. If a new trajectory is more optimal than the previous ones, the TQGR is calculated (Algorithm 1, Line 13). Then, the TQGR is assessed if the anytime planning (Algorithm 1, Line 3–16) needs to terminate its iteration (Algorithm 1, Line 15).

### 3.2 Quasi-dynamic vehicle model

**FIGURE 4:**
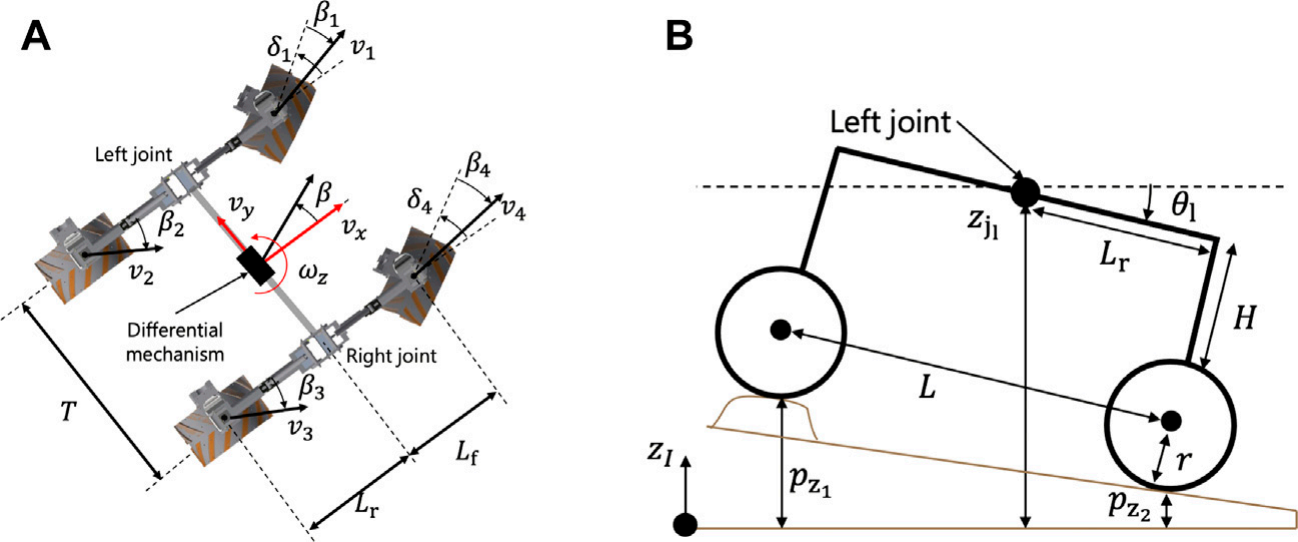
Schematic view of the vehicle model with a differential suspension. **(A)** Top view, **(B)** Side view.

The quasi-dynamic vehicle model was proposed in our conference article (Takemura and Ishigami, 2021). We validated that the planner with the quasi-dynamic model guarantees that the vehicle slippage is less than a prescribed threshold throughout the trajectory. This subsection only highlights the vehicle model since it is necessary to calculate the cost function. The rover considered in this study is assumed to be a four-wheeled mobile robot with a differential suspension and with front steerable wheels as shown in Figure 4. The proposed quasi-dynamic vehicle model contains five modules (Figure 3). First, the 2.5-dimensional kinematics with the vehicle slippage updates the vehicle state with regard to the vehicle position and heading. Subsequently, the suspension mechanism estimates vehicle roll and pitch on a sloped terrain. Given the roll and pitch angles, the wheel–soil interaction model based on terramechanics (Wong, 2008) calculates wheel contact forces for each wheel. Summing all contact forces provides the cornering and thrust of the vehicle. Additionally, for the summation, the commanded velocity and steering are given by the trajectory tracking controller. Using the cornering and thrust forces, the characteristic diagram of vehicle slippage (CDVS) estimates the vehicle slip ratio and sideslip angle. The following subsections carefully explain the five modules of the quasi-dynamic vehicle model.

#### 3.2.1 Kinematic formulation

The motion prediction on rough terrain is expressed based on the 2.5-dimensional kinematic vehicle model. First, a state of a rover is described as follows:

$$x={\left[x\;y\;\phi \;\theta \;\psi \;s\;\beta \right]}^{T},$$ (8)

where *x* and *y* are the coordinates of the center of gravity of the robot, and *ψ* is the heading angle of the robot. As in the study by Rajamani (2012), the robotic motion can be described in the robot’s body frame (Figure 4A) as follows:

$$v_{x}=v_{cmd}\left(1-s\right),$$ (9)

$$v_{y}=v_{x}\tan\beta ,$$ (10)

$$\omega_{z}=\frac{v_{x}\tan\delta }{L_{f}+L_{r}},$$ (11)

where *v*_*x*_ and *v*_*y*_ are the longitudinal and lateral velocities of the robot, respectively, *ω*_*z*_ is the heading rate of the robot, *L*_f_ and *L*_r_ are the distances from the center of gravity of the robot to the front and rear wheel axles, respectively, *v*_cmd_ is the command velocity along with the *x*-axis in the robot’s body frame, and *δ* is the steering angle of the front wheels of the robot. The steering angle is specified to the front wheels as a single scalar value, but it is appropriately decomposed to left and right steering angles based on the Ackermann steering geometry (Rajamani, 2012). *v*_cmd_ and *δ* are given by the trajectory tracking controller defined in Section 3.2.5.

The robot motion in the inertia frame is calculated by transforming the traveling velocity and heading rate in the robot’s body frame with the Euler angles (Howard and Kelly, 2007) as follows:

$$\left[\begin{array}{c} \dot{x}\\ \dot{y}\\ \dot{\psi } \end{array}\right]=R\left[\begin{array}{c} v_{x}\\ v_{y}\\ \omega_{z} \end{array}\right],$$ (12)

$$R=\left[\begin{array}{c} \cos\psi \cos\theta \;\cos\psi \sin\theta \sin\phi -\sin\psi \cos\phi \;0\\ \sin\psi \cos\theta \;\sin\psi \sin\theta \sin\phi +\cos\psi \cos\phi \;0\\ \;\;\;\;\;0\;\;\;\;\;\;\;\;\;\;\;\;\;\;\;\;0\;\;\;\;\;\;\;\;\;\;\;\;\cos\phi /\cos\theta \end{array}\right].$$

Additionally, the longitudinal and lateral velocities for each wheel $v_{x_{i}}$ and $v_{y_{i}}$ are calculated by the geometrical constraint:

$$\left[\begin{array}{c} v_{x_{i}}\\ v_{y_{i}}\\ v_{z_{i}} \end{array}\right]=\left[\begin{array}{c} v_{x}\\ v_{y}\\ 0 \end{array}\right]+\left[\begin{array}{c} 0\\ 0\\ \omega_{z} \end{array}\right]\times r_{i},$$ (13)

where the subscript *i denotes each wheel number, as shown in Figure 4A; ***r***_***i***_ is the vector from the center of gravity of the vehicle to that of each wheel. Focusing on a single wheel rotating on loose soil, the wheel experiences a certain amount of slippage that is quantified by the variables of a wheel slip ratio *s*_*i*_ and slip angle *β*_*i*_:

$$s_{i}=1-v_{x_{i}}/r\omega_{i},$$ (14)

$$\beta_{i}=\tan^{-1}\left(v_{y_{i}}/v_{x_{i}}\right),$$ (15)

where *r* is the radius of the wheel and *ω*_*i*_ is the angular velocity of the wheel, which is determined based on *v*_cmd_.

#### 3.2.2 Pose estimation

Once the position and the yaw angle are determined by the kinematic formulation, the roll and pitch angles are calculated. The calculation is based on the geometrical constraint between the differential suspension mechanism and terrain surface, as shown in Figure 4B. First, let us represent each wheel height for the four-wheeled rover as { $p_{z_{1}}$ , $p_{z_{2}}$ , $p_{z_{3}}$ , and $p_{z_{4}}$ }, as shown in Figure 4A. The height coordinate of each wheel is derived by a DEM node surrounding each wheel’s contact point. The roll and pitch angles are geometrically calculated as follows:

$$\phi =\arcsin\left(\frac{z_{j_{l}}-z_{j_{r}}}{T}\right),$$ (16)

$$\theta =\frac{\theta_{l}+\theta_{r}}{2},$$ (17)

where $z_{j_{l}}$ and $z_{j_{r}}$ are the heights of the left and right joints of the suspension system, respectively. *T* is the distance between the left and right joints. *θ*_l_ and *θ*_r_ denote the angles of the left and right joints, respectively, as shown in Figure 4B. $z_{j_{l}}$ and $z_{j_{r}}$ are calculated as follows based on the wheel contact points and geometric constraints of the rover:

$$z_{j_{l}}=p_{z_{2}}+\left(H+2r\right)\cos\theta_{l}-L_{r}\sin\theta_{l},$$ (18)

$$z_{j_{r}}=p_{z_{3}}+\left(H+2r\right)\cos\theta_{r}-L_{r}\sin\theta_{r},$$ (19)

where *H*, *r*, and *L*_r_ are the lengths defined in Figure 4B. *θ*_l_ and *θ*_r_ are given by the following equations:

$$\theta_{l}=\arcsin\left(\frac{p_{z_{2}}-p_{z_{1}}}{L}\right),$$ (20)

$$\theta_{r}=\arcsin\left(\frac{p_{z_{3}}-p_{z_{4}}}{L}\right),$$ (21)

where *L* is the wheelbase, which represents the length between the front and rear wheel axles.

#### 3.2.3 Wheel contact model based on terramechanics

**FIGURE 5:**
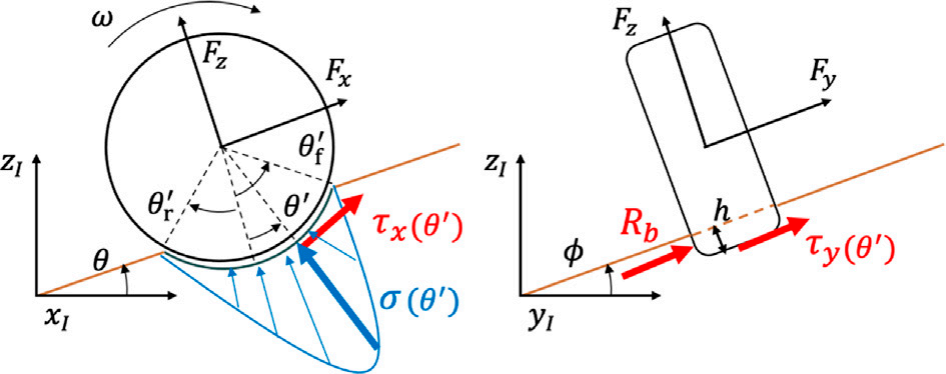
Wheel–soil contact force on a sloped terrain.

The wheel–soil interaction mechanics is described based on the Wong–Reece terramechanics model (Wong, 2008). Generally, wheels sink into loose soil while traveling on a slope, as shown in Figure 5. Beneath the wheels, the normal stress *σ* and the shear stress *τ*_{*x*,*y*}_ are distributed. A general force model for the *x*-axis $F_{x_{i}}$ , *y*-axis $F_{y_{i}}$ , and *z*-axis $F_{z_{i}}$ of each wheel is subsequently calculated as follows (Ishigami and Yoshida, 2005):

$$F_{x_{i}}=rb\int_{\theta_{r}^{\prime }}^{\theta_{f}^{\prime }}\left(\tau_{x}\left(\theta^{\prime }\right)\cos\theta^{\prime }-\sigma \left(\theta^{\prime }\right)\sin\theta^{\prime }\right.-R_{b}\left(\theta^{\prime }\right)\cos\beta_{i}d\theta^{\prime },$$ (22)

$$F_{y_{i}}=rb\int_{\theta_{r}^{\prime }}^{\theta_{f}^{\prime }}\left(\tau_{y}\left(\theta^{\prime }\right)+R_{b}\left(\theta^{\prime }\right)\sin\beta_{i}\right)d\theta^{\prime },$$ (23)

$$F_{z_{i}}=rb\int_{\theta_{r}^{\prime }}^{\theta_{f}^{\prime }}\left(\tau_{x}\left(\theta^{\prime }\right)\sin\left(\theta^{\prime }\right)+\sigma \left(\theta^{\prime }\right)\cos\theta^{\prime }\right)d\theta^{\prime },$$ (24)

where *R*_*b*_ is calculated based on Hegedus’s bulldozing resistance estimation (Hegedus, 1960), *b* is the wheel breadth, and $\theta_{f}^{\prime }$ and $\theta_{r}^{\prime }$ are the entry and exit angles, respectively. $\theta_{f}^{\prime }$ and $\theta_{r}^{\prime }$ are defined as follows:

$$\theta_{f}^{\prime }=\arccos\left(1.0-h/r\right),$$ (25)

$$\theta_{r}^{\prime }=-\arccos\left(1.0-\kappa h/r\right),$$ (26)

where *h* is the wheel sinkage and *κ* is the wheel sinkage ratio. Each wheel sinkage *h*_*i*_ is estimated by the following equation:

$$h_{i}=\underset{h}{\mathrm{argmin}}\left(F_{z_{i}}-W_{i}\left(\phi ,\theta \right)\right),$$ (27)

where *W*_*i*_ is each wheel load determined by the roll and pitch angles of the vehicle. Furthermore, the cornering and thrust forces are defined as sum of the wheel forces as follows:

$$F_{C}=\overset{n_{w}}{\underset{i=1}{\sum }}\left(F_{x_{i}}\sin\delta_{i}+F_{y_{i}}\cos\delta_{i}\right),$$ (28)

$$F_{T}=\overset{n_{w}}{\underset{i=1}{\sum }}\left(F_{x_{i}}\cos\delta_{i}-F_{y_{i}}\sin\delta_{i}\right),$$ (29)

where *n*_w_ is the number of wheels. These forces are highly related to the steering motion of the vehicle.

#### 3.2.4 Characteristic Diagram of Vehicle Slippage

**FIGURE 6:**
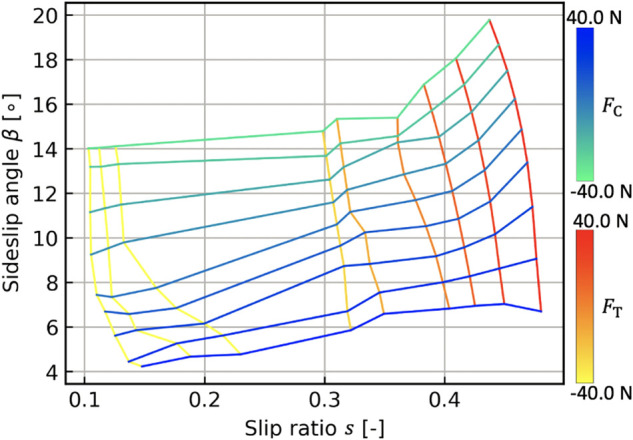
Characteristic diagram of vehicle slippage (*δ* =10°).

Our study assumes that the wheel slip effect in loose soil highly depends on the cornering and thrust forces of the vehicle. Hence, the vehicle dynamics and terramechanics elaborate the CDVS, which can fairly predict the vehicle slip motion in loose soil with less computational burden. To model the CDVS, first, a number of dynamic simulations (Ishigami and Yoshida, 2005) are conducted with different input values on the front steering angle and traction loads, while the target body velocity *v*_cmd_ for the vehicle is constant. The traction loads *F*_lat_ and *F*_lon_ are given in the lateral and longitudinal directions of the vehicle, which are the directions opposite to *v*_*y*_ and *v*_*x*_, respectively. The dynamic simulation outputs the wheel–soil interaction forces and the vehicle state variables such as *v*_*x*_ and *v*_*y*_. These output variables subsequently draw a two-dimensional diagram that varies *s* and *β* in accordance with *F*_C_, *F*_T_, and the steering angle, as illustrated in Figure 6. Once the CDVS is modeled by a regression approach such as support vector regression (Smola and Schölkopf, 2004) as offline processing, the vehicle slip ratio and the sideslip angle can be estimated based on the wheel–soil interaction forces. In the trajectory planning phase, *F*_C_, *F*_T_, and the front steering angle are used as an input to calculate *s* and *β* as follows:

$$s,\beta =g\left(F_{C},F_{T},\delta \right),$$ (30)

where the function *g* (⋅) expresses the modeled CDVS. The calculated *s* and *β* are directly exploited to update Eqs. 9, 10 in accordance with Figure 3. The quasi-dynamic vehicle model, therefore, can predict the vehicle state variables for robotic motions in loose soil with less computational burden as compared with the dynamic simulation (Ishigami and Yoshida, 2005). Similar approaches are reported by Sutoh et al. (2015) and Seegmiller and Kelly (2016), where wheel slippage is estimated based on the wheel–terrain interaction forces. The validity of the quasi-dynamic vehicle model using the CDVS is evaluated in our previous work (Takemura and Ishigami, 2021).

#### 3.2.5 Trajectory tracking controller

A trajectory tracking controller is realized by steering and driving actuators such that the robot can compensate for the wheel slip effect and smoothly follow reference trajectories within its maneuverability. The wheel slip compensation control proposed by Ishigami et al. (2009) is exploited. The desired steering control for the *i*-th wheel is expressed as follows:

$$\delta_{d_{\text{i}}}=\arctan\left(\frac{v_{d}\sin\psi_{d}-\dot{Y_{i}}\left({\dot{\psi }}_{d}\right)}{v_{d}\cos\psi_{d}-\dot{X_{i}}\left({\dot{\psi }}_{d}\right)}\right)-\psi_{d}-\beta_{i},$$ (31)

where *v*_d_ represents the desired linear velocity of the robot, and *X*_*i*_ and *Y*_*i*_ are the distances between the *i*-th wheel and the center of gravity of the robot in the *x* and *y* directions of the robot body frame, respectively. *ψ*_d_ represents the desired heading angle of the robot.

Subsequently, the command velocity compensating the longitudinal slip is written as

$$v_{cmd}=\frac{v_{d}}{1-s}.$$ (32)

In this study, the desired velocity *v*_d_ is expressed as follows:

$$v_{d}=v_{\min}+\frac{G}{G_{\max}}\left(v_{\max}-v_{\min}\right),$$ (33)

where *v*_max_ and *v*_min_ are the maximum and minimum traveling velocities, respectively, which are defined by the driving actuator’s limitation. *G* is the power generated by the robot. *G*_max_ is the maximum of *G*. The power generation was calculated using the method presented in Sakayori and Ishigami (2016). Eq. 34 indicates that the desired velocity decreases when the robot can potentially generate a large amount of power.

### 3.3 Traversability assessment

The new trajectory segment is examined based on the robot traversability. This assessment consists of two criteria, namely, posture angle and vehicle slippage, which are calculated based on the quasi-dynamic vehicle model. The roll and pitch angles should be less than their threshold angles throughout the trajectory. Additionally, the vehicle slippage including the wheel slip ratio and slip angle should be less than their threshold values. These threshold values can be predetermined by practical experiments such as slope traversability or mobility tests (Ishigami and Yoshida, 2005; Inotsume et al., 2012). This assessment limits the tree extension within the traversable regions on the terrain; hence, it guarantees that the trajectory is traversable for the vehicle in rough and loose terrains. It is noteworthy that the threshold value correlates safety of the trajectory and rover motion; larger values will generate a challenging trajectory with aggressive maneuvers for the rover, while smaller ones will generate a safe trajectory with a modest motion of the rover.

## 4 Terrain data processing

### 4.1 LiDAR-based 3D terrain mapping

**FIGURE 7:**
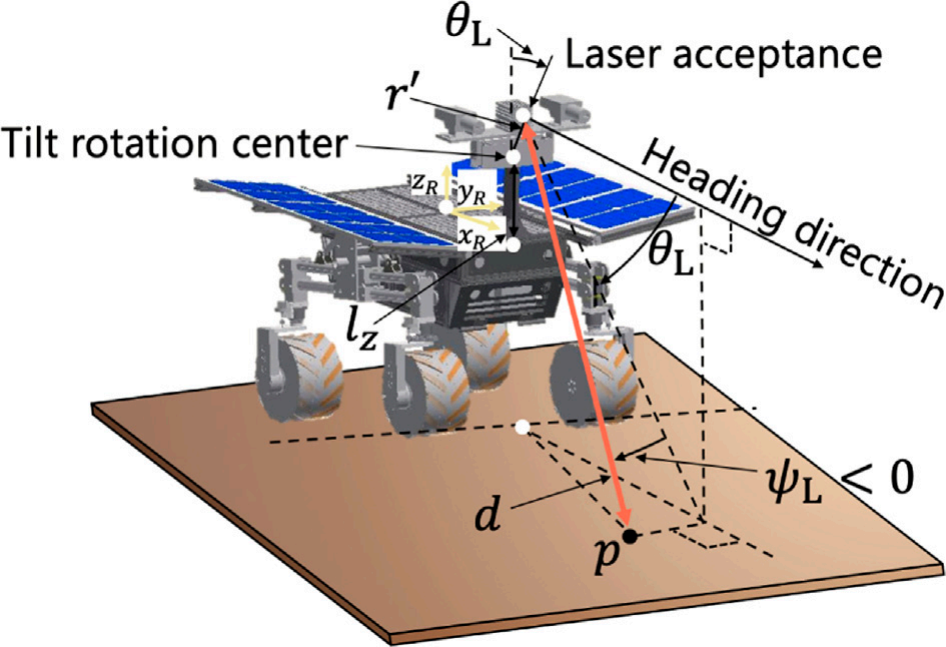
Geometrical analysis of LiDAR’s mapping.

To generate a DEM on real rough terrain, an experimental setup for a gimbaled LiDAR scanning system is first introduced. The plane-scanning LiDAR (UTM-30LX-EW developed by Hokuyo Corp.) is mounted on the gimbal, as shown in Figure 7, enabling the three-dimensional scanning for the terrain features. The laser emitter and acceptance point inside the LiDAR rotate 270° in the yaw direction, and then, the LiDAR can achieve the 2D plane scanning. Controlling the tilting motion of the LiDAR mounted on the gimbal along with the 2D plane scanning, 3D terrain mapping can be achieved. The LiDAR provides a 3D terrain feature as a dataset of a point cloud.

A geometrical analysis of the LiDAR system is illustrated in Figure 7. In this figure, one single point *p* is scanned by the LiDAR. Here, the coordinates of the point in the robot coordinate system *p*_*R*_ are given as follows:

$$p_{R}=r^{\prime }\left[\begin{array}{c} \sin\theta_{L}\\ 0\\ \cos\theta_{L} \end{array}\right]+d\left[\begin{array}{c} \cos\psi_{L}\cos\theta_{L}\\ \sin\psi_{L}\\ -\cos\psi_{L}\sin\theta_{L} \end{array}\right]+\left[\begin{array}{c} l_{x}\\ l_{y}\\ l_{z} \end{array}\right],$$ (34)

where the offset distance between the tilt rotation center and the light acceptance point is represented by *r*′, *d* is the distance from the LiDAR to the point, *ψ*_L_ is the scanning angle around the yaw of the LiDAR, *θ*_L_ is the tilting angle with 0.0° being horizontal, and *l*_*x*_, *l*_*y*_, and *l*_*z*_ are the distances between the center of gravity of the robot and the tilt rotation center in *x*, *y*, and *z* directions of the robot body frame, respectively. *p*_*R*_ needs to be transformed from the robot coordinate system to an inertial coordinate system based on a rotation matrix. The matrix is composed of the robot configuration: roll, pitch, and yaw angles can be measured by an onboard IMU. The inertial coordinate *p*_*I*_ is given by the rotation matrix of the Euler angles as follows:

$$p_{I}=R_{z}R_{y}R_{x}p_{R}.$$ (35)

Then, a DEM is generated by downsampling the number of point cloud data, as presented in Ishigami et al. (2013) and Rekleitis et al. (2013). The DEM represents terrain elevations for ground positions at regularly spaced intervals.

### 4.2 Experiment for terrain map acquisition

**FIGURE 8:**
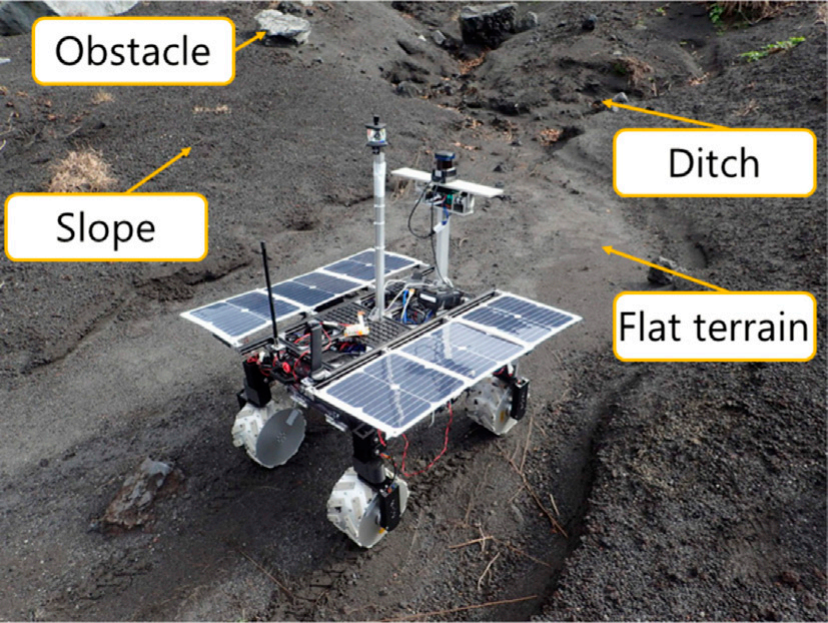
Gimbaled LiDAR scanning system for 3D terrain mapping.

**FIGURE 9:**
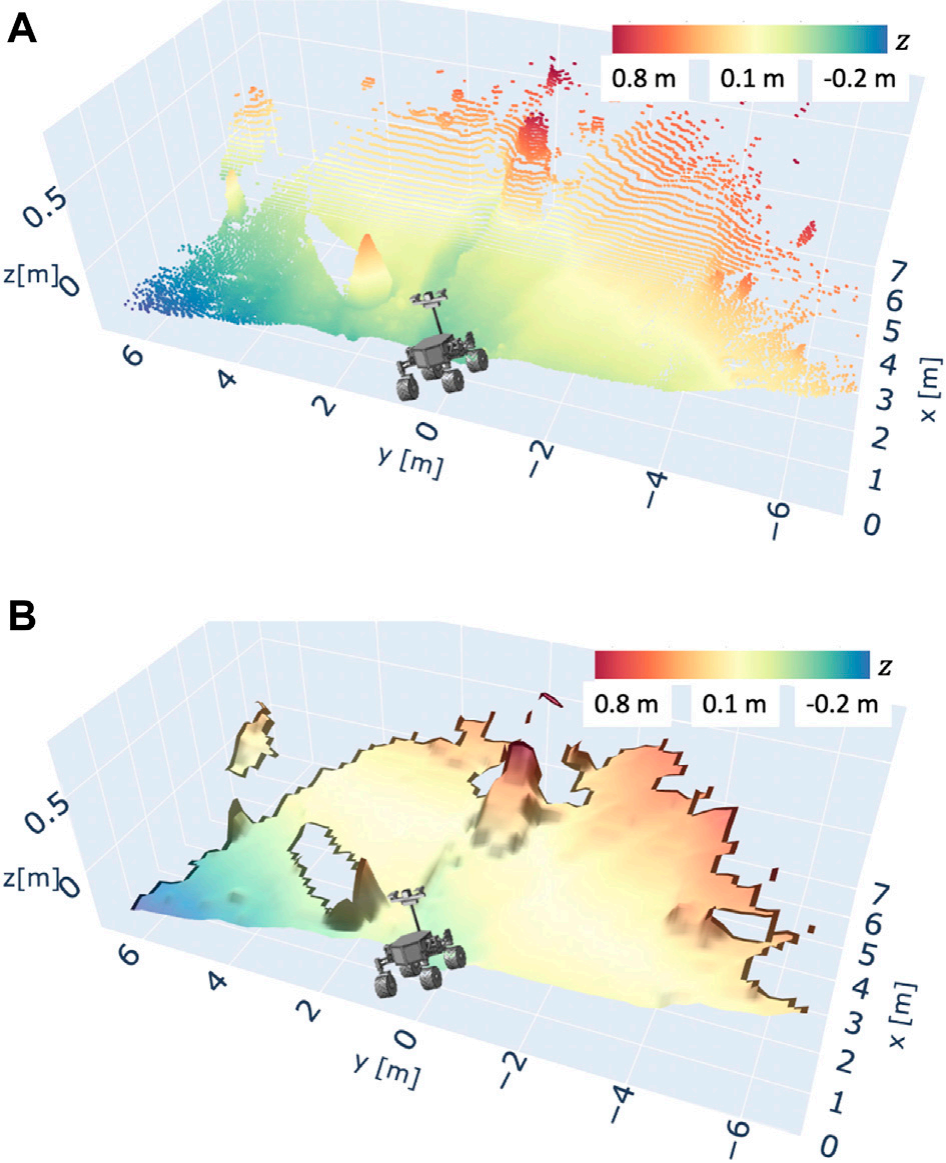
Example of a 3D terrain map scanned by LiDAR. The terrain surface is scanned from the location of the illustration of the rover testbed. **(A)** Raw point cloud data scanned by LiDAR, **(B)** DEM converted from the raw point cloud dataset.

The experiment of terrain mapping was conducted in a volcanic area at Mt. Mihara, Japan. In this area, the terrain is mainly covered with dark volcanic basalt rocks called scoria. Additionally, the terrain feature is mostly composed of slopes, ditches, and volcanic bombs (huge rocks), as shown in Figure 8. In the experiment, first, the robot posture was measured by the IMU at scanning positions. The LiDAR scanning starts from 0 to 80° in the tilt axle of the gimbal unit. The range of *ψ*_*L*_ is given as [ − 90.0°, 90.0°]. The LiDAR provides 721 points in every line scanning and repeats this line scanning about 150 times while the gimbal rotates. Therefore, one complete 3D scanning contains about 100, 000 points. The LiDAR used in this experiment can usually measure a distance of about 30 m. However, the scoria terrain barely reflects the light emitted from the LiDAR, and the reflected light becomes weaker as the distance gets further. Therefore, the work in this article assumes that the data obtained from 0.0 to 6.0 m are reliable enough to be exploited for DEM generation. Figure 9 shows the typical result of the LiDAR scanning experiment and the conversion to a DEM.

### 4.3 Terrain classifier

The LiDAR scanning data are classified based on terrain roughness for the statistical analysis of the TQGR. The terrain roughness is defined as follows:

$$B=\sqrt{\frac{1}{n_{p}}\overset{n_{p}}{\underset{i=0}{\sum }}{\left(z_{I_{i}}-\bar{z_{I}}\right)}^{2}},$$ (36)

where *B* is the roughness of the area with the tree expanded by the planner, $z_{I_{i}}$ is the height of the point cloud data, $\bar{z_{I}}$ is the average of $z_{I_{i}}$ , and *n*_*p*_ is the number of the point cloud.

Another method to classify surrounding terrain types is to use machine learning-based approaches (Filitchkin and Byl, 2012; Rothrock et al., 2016; Higa et al., 2019; Iwashita et al., 2019). These works have achieved the terrain classification to accurately predict vehicle slippage or estimate energy consumption. Given that detailed terrain classification is not the core of our contribution here, we used Eq. 37 in our planning framework. For instance, Figure 9 shows terrain roughness 0.149 m.

## 5 Simulation study

In this study, offline and online trajectory planning simulations are conducted. The offline planning models the TQGR-based analysis module, and the online use case validates the performance of the TQGR-based trajectory planning framework. Simulation parameters are summarized in Table 1.

**TABLE 1:** Parameters used in the simulation.

Parameter	Value	Unit
*ϕ*_th_, *N*_*ϕ*_	20.0	Degrees
*θ*_th_, *N*_*θ*_	20.0	Degrees
*s*_th_, *N*_*s*_	0.90	-
*β*_th_, *N*_*β*_	45.0	Degrees
*N*_*L*_	1.0	M
*W*_*L*_	0.20	-
*W*_*ϕ*_, *W*_*θ*_	0.30	-
*W*_*s*_	0.05	-
*W*_*β*_	0.15	-
*E*_max_	700	S
*α*	0.9	-
*L*_f_, *L*_r_	0.30	M
*L*	0.60	M
*T*	0.50	M
*H*	0.30	M
*r*	0.10	M
*m*	38.5	kg

### 5.1 Offline trajectory planning for learning TQGR-based analysis

**FIGURE 10:**
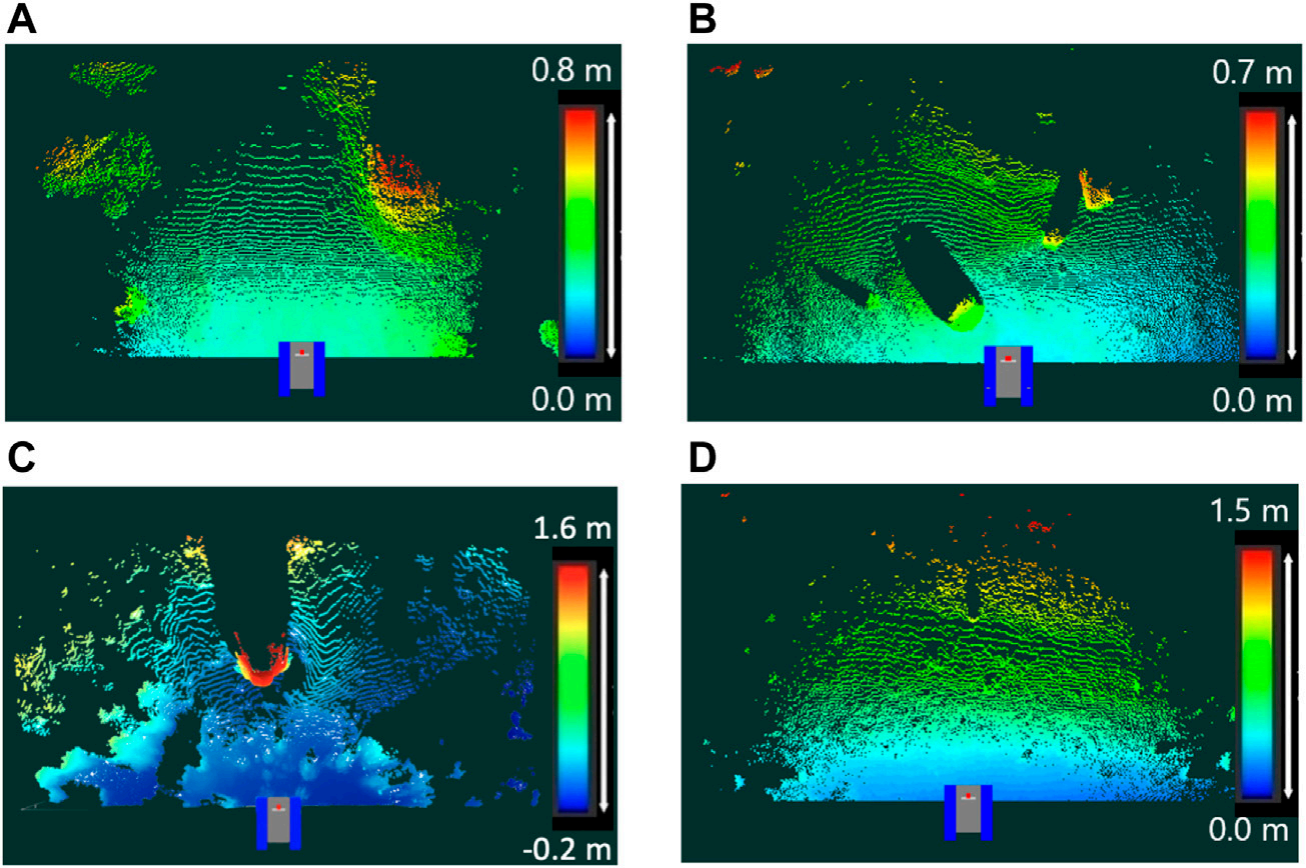
Typical terrain maps for simulation. The initial position is set at the scanning point illustrated by the 2D model of the rover testbed. **(A)** Flat terrain: roughness 0.083 m, **(B)** Rocky terrain: roughness 0.123 m, **(C)** Rough terrain: roughness 0.234 m, **(D)** Sloped terrain: roughness 0.257 m.

**FIGURE 11:**
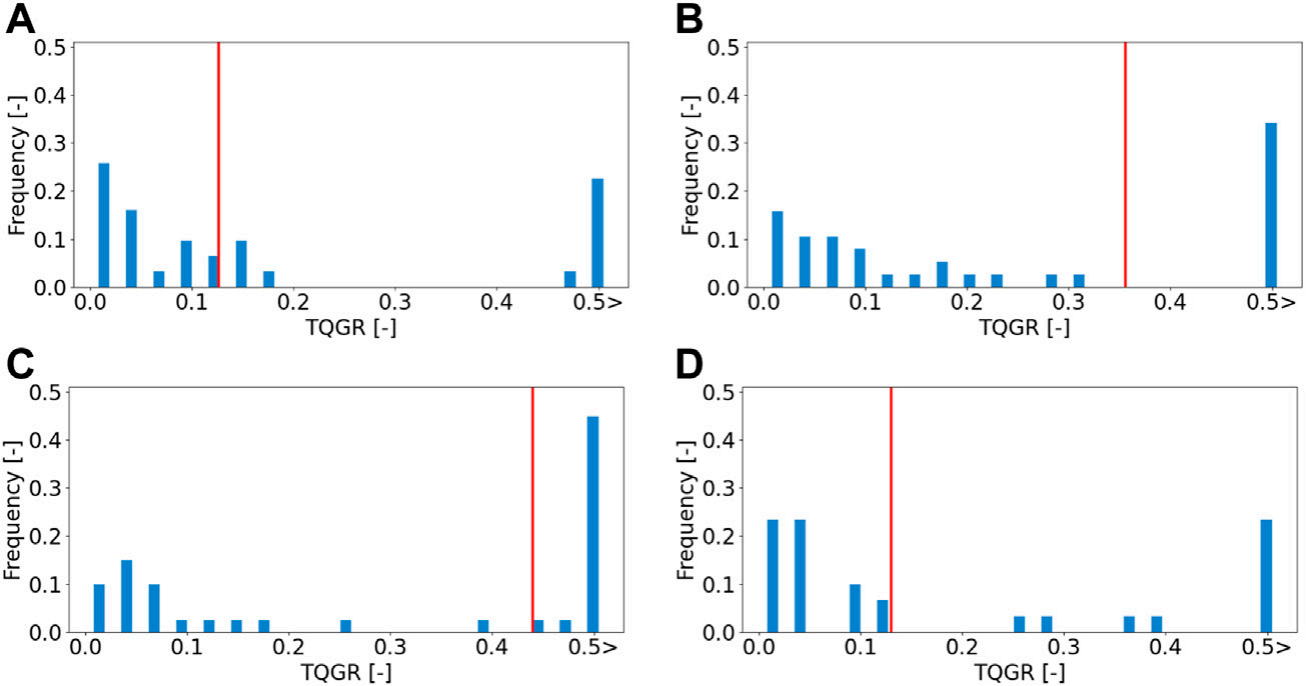
Histogram of the TQGRs for each terrain. Red lines show the geometric mean of TQGRs. **(A)** Flat terrain: roughness 0.083 m, **(B)** Rocky terrain: roughness 0.123 m, **(C)** Rough terrain: roughness 0.234 m, **(D)** Sloped terrain: roughness 0.257 m.

We obtained 11 types of terrain data through the field experiment in Section 4.2. For the statistical analysis of the TQGR, nine of the 11 datasets were exploited so that the terrain roughness is widely distributed. Four typical terrain maps are shown in Figure 10. For each terrain, two or three goals were randomly sampled within a range from 5.0 to 6.0 m, and we defined a set of one goal and one terrain as a scenario. Each initial position was set in the LiDAR scanning point, as shown in Figure 10. The anytime trajectory planning was repeatedly executed 20 times for each scenario. Each repetition has 15 iterations of the anytime planning. Typical results of TQGRs are summarized in the histogram illustrated in Figure 11.

According to the results, we observed that the TQGR was frequently divided into small (<0.2) or large (0.5 >) values in all terrain types. This implies that the improvement of the trajectory quality occurs either exceedingly or slightly. Given the geometric mean *q* for each scenario, the trend of the TQGR is separated into two groups, namely, flat/sloped terrains and rocky/rough terrains. The first group acquired a smaller *q*, and the second group acquired the larger one. We deduce this because the trajectory quality varies scarcely with lower local terrain roughness. The cost function (Eq. 7) mainly consists of rover posture angles and slippage; therefore, flat and sloped terrains have a low expectation to reduce the cost. On the other hand, in rocky and rough terrains, the planner can frequently improve trajectory quality; hence, a larger *q* is obtained.

**FIGURE 12:**
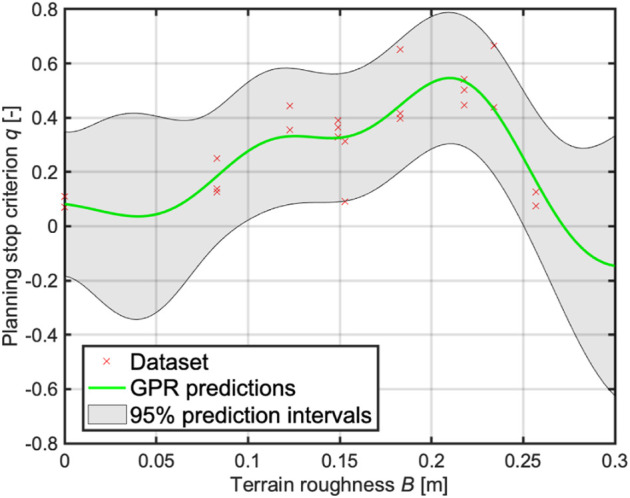
GPR model for the TQGR-based analysis module, which shows the terrain roughness vs. the planning stop criterion.

As described, *q* seems to correlate with the terrain features. Then, the GPR algorithm generates the TQGR-based analysis module by modeling the relationship between the terrain roughness and the planning stop criterion, as shown in Figure 12. The GPR not only outputs the predicted value but also 95% confidence intervals. As the characteristics of GPR, the prediction accuracy is worse where there is no dataset. In this study, the upper and lower boundaries are defined as *q* ^+^ and *q* ^−^, respectively.

### 5.2 Online trajectory planning scenario

**FIGURE 13:**
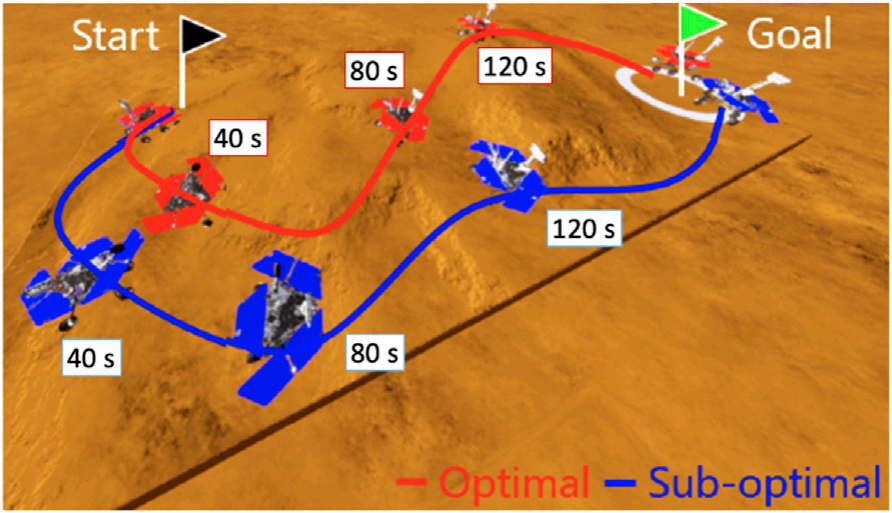
Illustration of the difference between the sub-optimal and nearly optimal trajectories. The two trajectories are quite different, but the costs are almost the same because of the rough and loose terrains.

**FIGURE 14:**
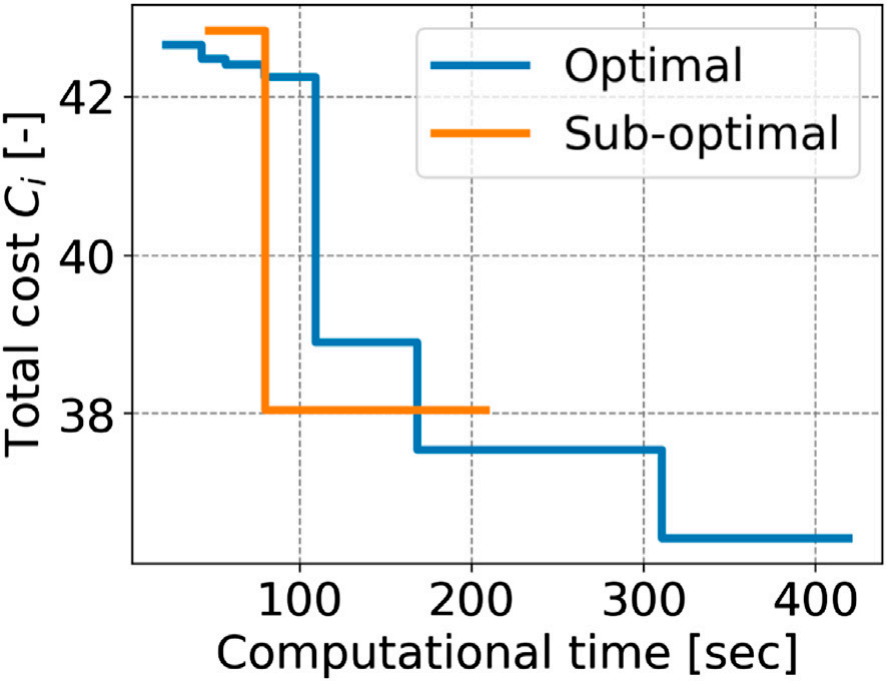
Time history of computational time vs. cost.

To validate the effectiveness of the proposed planning stop criterion in online planning scenarios, the sub-optimal trajectory planning is compared with the nearly optimal planning in LiDAR-based terrain maps. Two typical terrain maps were used, and goals greater than 5 m (A and B) were selected for the trajectory planning simulation. The terrain maps are given by the LiDAR-based mapping in Section 4.2, but they are not used for GPR modeling. Terrain roughnesses are 0.207 and 0.281 [m], and their *q* values are predicted as shown in Table 2 and used for the sub-optimal trajectory planning. It should be noted that it needs too much computational time to find the optimal trajectory; hence, the trajectory found at the 15-th iteration is defined as a nearly optimal solution. To consider the randomness of the planner, 20 trials of trajectory planning simulation are conducted. The proposed trajectory planning framework was implemented in Python and ran on a computer with an Intel i5 CPU 2.0 GHz processor and 16 GB RAM. The typical result of the sub-optimal and nearly optimal trajectories is illustrated in Figure 13. The typical time history of the total cost is shown in Figure 14. Tables 3–6 summarize the average and standard deviation throughout the 20 trials for computational time, final cost, and its improvement rate. The cost improvement *CI* is calculated as follows:

$$CI=1-\frac{C_{\mathrm{fi}n}}{C_{0}},$$ (37)

where *C*_fin_ is the final cost.First, we observed that both total costs largely decreased until about 100 s, as shown in Figure 14. The cost of the sub-optimal trajectory is 38.1, resulting in a difference of only 4.3% from the nearly optimal one. Subsequently, the proposed planner was terminated at 208 s, while the nearly optimal trajectory was found around 310 s and its planner continues around 420 s. This means that the TQGR-based analysis module appropriately outputs the planning stop criterion, enabling the planner to save the computational cost by 50.5%. We observed the nearly optimal trajectory conducted useless iterations because the TQGR around 200 s is relatively low. Therefore, we can conclude the proposed planner avoided the useless calculation.

**TABLE 2:** Planning stop criterion.

Roughness	*q* ^−^	*q*	*q* ^+^
0.207	0.302	0.545	0.788
0.281	−0.435	−0.074	0.288

**TABLE 3:** Simulation result in terrain roughness 0.207 (goal A): *C*_0_=43.0 ± 3.1.

Trajectory	Time [s]		Final cost [-]		Cost imp. [%]	
	Ave	Std	Ave	Std	Ave	Std
Sub-optimal: *q* ^+^	127.7	37.3	42.1	3.1	2.0	3.1
Sub-optimal: *q*	209.2	90.9	40.7	2.4	5.0	6.5
Sub-optimal: *q* ^−^	373.7	111.1	39.5	2.3	7.7	7.3
Optimal	587.2	89.8	38.1	1.6	10.9	7.0

**TABLE 4:** Simulation result in terrain roughness 0.207 (goal B): *C*_0_=39.6 ± 2.1.

Trajectory	Time [s]		Final cost [-]		Cost imp. [%]	
	Ave	Std	Ave	Std	Ave	Std
Sub-optimal: *q* ^+^	154.9	53.4	38.5	1.8	2.7	3.9
Sub-optimal: *q*	216.8	78.6	37.8	1.1	4.3	5.4
Sub-optimal: *q* ^−^	409.2	129.5	37.2	1.0	5.9	6.0
Optimal	586.8	92.5	37.0	0.9	6.4	5.7

**TABLE 5:** Simulation result in terrain roughness 0.281 (goal A): *C*_0_=90.9 ± 1.7.

Trajectory	Time [s]		Final cost [-]		Cost imp. [%]	
	Ave	Std	Ave	Std	Ave	Std
Sub-optimal: *q* ^+^	455.4	100.1	88.2	1.6	2.9	2.3
Sub-optimal: *q*	-	-	-	-	-	-
Sub-optimal: *q* ^−^	-	-	-	-	-	-
Optimal	531.3	56.7	87.9	1.3	3.2	2.1

**TABLE 6:** Simulation result in terrain roughness 0.281 (goal B): *C*_0_=92.1 ± 3.1.

Trajectory	Time [s]		Final cost [-]		Cost imp. [%]	
	Ave	Std	Ave	Std	Ave	Std
Sub-optimal: *q* ^+^	446.0	106.2	88.8	2.4	3.5	2.9
Sub-optimal: *q*	-	-	-	-	-	-
Sub-optimal: *q* ^−^	-	-	-	-	-	-
Optimal	557.8	79.1	88.1	1.7	4.2	3.3

Based on the results in Tables 3–6, two metrics were calculated to evaluate the performance of the proposed framework:

• Time improvement ratio: how much time is improved as compared with the nearly optimal planner? This can be calculated as follows:

$$I_{t}=100\left(1-\frac{t_{q^{*}}}{t_{opt}}\right),$$ (38)

where *t* ^.^ is each average time in Tables 3–6, and q* is replaced by *q*, *q* ^+^, and *q* ^−^. It is noted that we evaluated the planner using *I*_*t*_ instead of computational time since our implementation does not assume the CPU power and programming language required for actual operation.

• Cost improvement ratio: how much better *CI* is as compared with the nearly optimal planner? This can be calculated as follows:

$$I_{C}=100\left(\frac{CI_{q^{*}}}{CI_{opt}}\right),$$ (39)

where *CI* ^.^ is each cost improvement in Tables 3–6.

**FIGURE 15:**
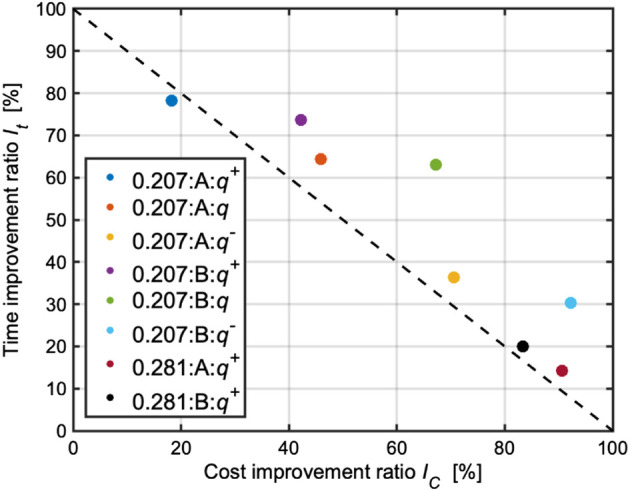
Summary of two metrics (*I*_*C*_ vs. *I*_*t*_) in the simulation results. The legend shows a set of terrain roughness, goal, and planning stop criterion. The dashed line describes the benchmark of the relationship between *I*_*C*_ and *I*_*t*_. For instance, if the trajectory planning stops at an 80% cost improvement of the nearly optimal trajectory planner, it stands to reason that a 20% time improvement would be observed.

Figure 15 shows two metrics for each terrain map and each goal and the benchmark for the evaluation.

In the terrain with 0.207 [m] roughness, the sub-optimal trajectory planning with *q* observed cost improvements of 45.9% and 67.2% compared with the nearly optimal planning. Additionally, the sub-optimal planner improved computational time by 64.4% and 63.1%. These results show the sub-optimal trajectory planning with *q* terminated the planning iteration while exceeding the benchmark. The cases for *q* ^+^ and *q* ^−^ still outperformed the benchmark except for *q* ^+^ in goal A. It is noted that the deterioration in *q* ^+^ in goal A is only 3.4%. This point would be improved if the GPR modeling is elaborated further by an increase of the dataset.

In the terrain with 0.281 [m] roughness, *q* and *q* ^−^ are minus values; hence, Eq. 5 does not work well. This is because this terrain roughness is an extrapolation for the GPR modeling, resulting in a less accurate prediction. According to Figure 15, we observed that the computational times of the case with *q* ^+^ were only reduced by 14.3% and 20.0%, while their cost improvements were 90.6% and 83.3%. The performance of *q* ^+^ did not significantly exceed the benchmark as compared with the case for 0.207 [m] roughness. This point would also be improved if the number of dataset for the GPR model increases.

Overall, we observed an average reduction in a computational time of 47.6%, with a cost improvement of 63.8%. Therefore, the TQGR-based analysis module could suggest the appropriate time to terminate the iteration of the anytime planning for each terrain type, resulting in less computational burden. The problem of the lack of dataset would be solved by an informative motion planning algorithm (Viseras et al., 2017), which allows the rover to identify unexplored environments. This approach contributes to efficient data collection for the GPR modeling, and this issue will be addressed as a future work.

## 6 Conclusion and future work

This study introduced TQGR analysis, which contributes to solving the trade-off problem between the optimality and computational burden for the incremental search algorithm. The TQGR-based trajectory planning framework can appropriately terminate its planning for each type of terrain, enabling the rover to generate a sub-optimal trajectory with less computational burden. The TQGR analysis module was modeled based on multiple trials of trajectory simulation in a real rough terrain. Our statistical analysis of the TQGR revealed that the planning stop criterion correlates with the terrain features. The GPR algorithm models the relation, and the trajectory planning simulation in unknown environments confirmed that on average, the proposed framework can reduce the computational cost by 47.6% while maintaining 63.8% of trajectory optimality. Even though the TQGR-based analysis module could not adequately predict the planning stop criterion, the proposed framework still worked better than the benchmark.

A possible future direction of this study is efficient data collection to improve the accuracy of the TQGR-based analysis module. As discussed, the informative motion planner efficiently explores unknown environments, which would contribute to the collection of the useful dataset for GPR modeling.

Another future work possibly includes the implementation of an asymptotically optimal algorithm such as RRT* (Karaman and Frazzoli, 2011) instead of the anytime RRT algorithm. This may efficiently find a cost-minimum trajectory, and the planning stop criterion possibly performed better. However, the original RRT* algorithm needs to consider an appropriate steering function for a wheeled robot model. As reported in Jeon and Frazzoli (2011), this two-point boundary value problem is computationally inefficient, and this is why we did not use RRT* in this study. Hence, we would need another approach such as CL-RRT^*#*^, which does not require solving the steering maneuver problem (Arslan and Tsiotras, 2017).

## Data Availability

The raw data supporting the conclusions of this article will be made available by the authors, without undue reservation.
